# Intracardiac Hemostasis and Fibrinolysis Parameters in Patients with Atrial Fibrillation

**DOI:** 10.1155/2017/3678017

**Published:** 2017-06-21

**Authors:** Noémi Klára Tóth, Zoltán Csanádi, Orsolya Hajas, Alexandra Kiss, Edina Nagy-Baló, Kitti Bernadett Kovács, Ferenc Sarkady, László Muszbek, Zsuzsanna Bereczky, László Csiba, Zsuzsa Bagoly

**Affiliations:** ^1^Division of Clinical Laboratory Sciences, Faculty of Medicine, University of Debrecen, Debrecen, Hungary; ^2^Institute of Cardiology, Faculty of Medicine, University of Debrecen, Debrecen, Hungary; ^3^Department of Neurology, Faculty of Medicine, University of Debrecen, Debrecen, Hungary

## Abstract

**Aims:**

To identify intracardiac hemostasis or fibrinolysis abnormalities, which are associated with atrial fibrillation (AF) and increase the risk of thromboembolism.

**Patients and Methods:**

Patient group consisted of 24 patients with AF and control group included 14 individuals with other supraventricular tachycardia undergoing transcatheter radiofrequency ablation. Blood samples were drawn from the femoral vein (FV), left atrium (LA), and left atrial appendage (LAA) before the ablation procedure. Fibrinogen, factor VIII (FVIII) and factor XIII activity, von Willebrand factor (VWF) antigen, thrombin-antithrombin (TAT) complex, quantitative fibrin monomer (FM), plasminogen, *α*_2_-plasmin inhibitor, plasmin-*α*_2_-antiplasmin (PAP) complex, PAI-1 activity, and D-dimer were measured from all samples.

**Results:**

Levels of FVIII and VWF were significantly elevated in the FV and LA of AF patients as compared to controls. TAT complex, FM, PAP complex, and D-dimer levels were significantly elevated in the LA as compared to FV samples in case of both groups, indicating a temporary thrombotic risk associated with the catheterization procedure.

**Conclusions:**

None of the investigated hemostasis or fibrinolysis parameters showed significant intracardiac alterations in AF patients as compared to non-AF controls. AF patients have elevated FVIII and VWF levels, most likely due to endothelial damage, presenting at both intracardiac and systemic level.

## 1. Introduction

Atrial fibrillation (AF) is the most common sustained cardiac arrhythmia associated with a high risk of mortality and morbidity from stroke and thromboembolism [1]. The precise mechanism by which AF causes thromboembolism and subsequent cerebrovascular events is yet to be fully elucidated. It is widely recognized that thromboembolism in AF is associated with a combination of pathophysiological mechanisms, which fulfill the requirement of Virchow's triad for thrombogenesis: stasis, abnormal change in the vessel wall and pathological imbalance of hemostasis, and fibrinolysis [2]. Although several risk factors (age, hypertension, diabetes mellitus, etc.) predispose to stroke in patients with AF, it has been controversial whether the arrhythmia itself leads to hypercoagulability [3–5]. Nonetheless, it is a well-known fact that anticoagulation therapy reduces the risk of ischemic stroke in AF patients by two-thirds [6]. There are a number of reports available on the activation of coagulation cascade in AF, but fibrinolytic activity in AF patients has been less studied [7–10]. Most studies have focused on the relation of AF-associated thromboembolism with various endothelial damage markers, prothrombotic or inflammatory factors, and plasma markers of platelet activation [4, 8, 11, 12]. It has been shown that AF is associated with increased levels of prothrombin fragments 1 + 2 (F1 + 2) and thrombin-antithrombin (TAT) complex, elevated plasma fibrinogen levels alongside with endothelial markers of soluble thrombomodulin, and von Willebrand factor (VWF) [2, 13–16]. The role of fibrinolysis in the pathomechanism of AF-associated thromboembolism has been much less studied and controversial findings have been published. AF has been associated with hypofibrinolysis due to increased levels of plasminogen activator inhibitor type 1 (PAI-1) [17], although in other studies hyperfibrinolysis with elevated tissue plasminogen activator (t-PA) [18–22] and plasmin-*α*_2_-antiplasmin (PAP) complex levels were found [23]. Impaired fibrinolysis due to unfavorably altered fibrin clot properties has been also described in AF patients [9]. These reports present data on coagulation and fibrinolytic markers measured in peripheral samples; however, the prothrombotic effects of AF are most likely to develop on a local, cardiac level and may not manifest in the peripheral circulation. Recent studies suggest that there is a difference between the left atrium (LA) and the systemic circulation in AF patients and the thrombogenic pathology is more likely to be restricted to the heart [24, 25]. However, most possibly due to the difficulty of intracardiac blood sampling, in the last decade only few studies, involving a relatively small number of patients, investigated the hemostasis or fibrinolytic system in samples obtained from the LA of patients with AF [11, 26–28]. Unfortunately, these few studies using LA samples published so far examined only a subset of coagulation markers and even less is known about the fibrinolytic system in this respect. As to our knowledge, the levels of blood coagulation factor XIII (FXIII) or *α*_2_-plasmin inhibitor (*α*_2_-PI), although key regulators of fibrinolysis, have not been studied from intracardiac samples as yet. Moreover, in most cases, results obtained from the intracardiac samples of AF patients were not compared to age and sex-matched non-AF intracardiac samples [11, 26]. As the intervention of intracardiac blood sampling is an invasive procedure with a potential effect on the hemostasis system, it is essential to compare results to a population undergoing the same intervention, but not having AF. Furthermore, in some studies patients received unfractionated heparin prior intracardiac blood sampling, which of course has a major impact on the hemostasis system and limits the number of parameters that could be determined from these samples [26]. The investigation of blood samples obtained from the left atrial appendage (LAA) of AF patients is also intriguing, as it has been suggested that the LAA is the most thrombogenic part of the heart in AF, being the most frequent location of embolic thrombi [29]. Nevertheless, available data on coagulation or fibrinolysis markers in LAA samples of AF patients are a rarity.

In this study we aimed to identify local hemostasis and fibrinolysis abnormalities, which are associated with AF and increase the risk of thromboembolism. Intracardiac blood samples taken from the LA and LAA of AF patients and non-AF controls were tested for a comprehensive set of hemostasis and fibrinolytic factors in order to assess AF-associated alterations.

## 2. Methods

### 2.1. Study Population

Consecutive patients undergoing radiofrequency ablation for symptomatic paroxysmal or persistent AF (AF group) as well as age- and sex-matched patients with any arrhythmia other than AF requiring left atrial access (non-AF control group) were enrolled in the study. Patients were enrolled between 2013 October and 2015 December. All AF patients were undergoing pulmonary vein isolation (PVI) with phased radiofrequency (RF) or cryoballoon ablation procedure. Non-AF controls were undergoing routine RF ablation of a left atrial substrate (mostly a left-sided accessory atrioventricular pathway).

Inclusion criteria for the AF group were the following: age 18–75 years, documented, symptomatic paroxysmal or persistent AF, failure of at least one antiarrhythmic drug, and patient being willing to sign a written informed consent. Inclusion criteria for the control group were age 18–75 years, documented non-AF arrhythmia including one of the following: left atrial tachycardia, paroxysmal supraventricular tachycardia (orthodromic or antidromic), or FBI (fast, broad, and irregular) tachycardia due to a left-sided accessory pathway, preexcitation on the 12-lead electrocardiogram in an asymptomatic individual in whom the electrophysiology study revealed a left-sided accessory pathway potentially resulting in significant arrhythmia based on its conduction properties, and patient being willing to sign a written informed consent. Exclusion criteria for the patient and control groups were previous heart surgery, valvular heart disease, left ventricular ejection fraction (LVEF) ≤ 30%, heart failure of New York Heart Association functional classification (NYHA) class III or IV, documented carotid stenosis, history of ischemic stroke or TIA, prior cardiac surgery, unstable angina or myocardial infarction within the last 3 months, severe chronic obstructive pulmonary disease, known bleeding or thrombotic disorders, acute inflammation, contraindication to oral anticoagulation or to diffusion weighted magnetic resonance imaging (DW MRI), and pregnancy. Additional exclusion criteria for the patient group were long-standing persistent AF, reversible cause of AF (e.g., hyperthyroidism), and presence of AF thrombus.

Risk factors for stroke (hypertension, diabetes mellitus, smoking, BMI, etc.) together with the list of current medications were assessed before the enrollment of patients. The CHADS_2_ score (congestive heart failure, hypertension, age ≥ 75 years, diabetes mellitus, and stroke/transient ischemic attack), CHA_2_DS_2_-VASC score (congestive heart failure, hypertension, age ≥ 75 years, diabetes mellitus, stroke/transient ischemic attack/thromboembolism, vascular disease (prior myocardial infarction, peripheral vascular disease, or aortic atherosclerosis), age (65–74 years), and sex category (female)), and EHRA score (European Heart Rhythm Association score) [30] were recorded for every AF patient.

The study design was in accordance with the guiding principles of the Declaration of Helsinki, and was approved by the Institutional Ethics Committee of the University of Debrecen and the Ethics Committee of the National Medical Research Council (ETT-TUKEB). All patients signed a written informed consent form prior to inclusion.

### 2.2. Electrophysiology Procedure and Blood Drawing

Patients were hospitalized 1 or 2 days before the procedure. All medications with a potential effect on coagulation or platelet activity were discontinued for a period of at least three half-lives (or a period needed for reaching complete decay of their action) before the procedure. Transesophageal echocardiography was carried out within 24 h prior to the procedure in order to rule out the presence of a cardiac thrombus in all AF patients. All procedures were carried out under conscious sedation, using midazolam and fentanyl. The ablation procedures were performed as described previously [31, 32]. Blood samples were taken before the ablation procedures from multiple sites: (1) peripheral femoral venous (FV) sheath, (2) left atrial (LA) sheath, and (3) left atrial appendage (LAA) sheath. Intracardiac blood samples were collected before the administration of unfractionated heparin.

Briefly, three punctures of the right femoral vein were performed using the Seldinger technique and introducers with side arms were placed in the vein. Forty-five ml blood sample was drawn through the side arm of a short introducer immediately after access to the vein, from which the first 5 ml of blood was discarded in order to exclude intrasheath hemostasis activation (FV sample). Blood samples were collected into vacutainer tubes (tubes anticoagulated with K_3_-EDTA for complete blood count, tubes containing 0.109 M sodium citrate and CTAD (buffered citrate, theophylline, adenosine, and dipyridamole)) for hemostasis and fibrinolysis tests (Becton Dickinson, Franklin Lakes, NJ). After blood drawing, a decapolar catheter and an intracardiac echo (ICE) catheter were advanced from the femoral vein and positioned in the coronary sinus and in the right atrium, respectively. A single ICE-guided transseptal puncture was performed using a Mullins transseptal sheath and a Brockenborough needle (Medtronic, Kirkland, QC, Canada) under fluoroscopic and ICE guidance using standard technique. After crossing the septum, the dilator of the Mullins sheath was removed and 45 ml blood sample was drawn from the LA, from which the first 5 ml of blood was discarded (LA sample). LA blood samples were collected into vacutainer tubes as described above. After the blood drawing of LA samples, the LAA was accessed by using a 5 F pigtail catheter (Medtronic, Kirkland, QC, Canada) under fluoroscopy and ICE control. A blood sample of 45 ml was taken from the LAA, of which, again, the first 5 ml was discarded (LAA sample). LAA blood samples were collected into vacutainer tubes as described above. Immediately after blood samplings, 150 IU/kg body weight i.v. heparin was administered and ablations were performed according to standard protocols.

### 2.3. Laboratory Investigations

Blood samples anticoagulated with K_3_-EDTA were immediately tested for complete blood count. Blood samples anticoagulated with citrate or CTAD were centrifuged twice at 1500 g at room temperature for 20 min and plasma samples were stored at −70°C until further analysis. The measurement of plasminogen activator inhibitor-1 (PAI-1) activity was performed from plasma samples anticoagulated with CTAD; besides this measurement, all hemostasis and fibrinolysis tests were performed using citrated plasma. Hemostasis and fibrinolysis tests were performed from all sample types (FV, LA, and LAA samples). Screening tests of hemostasis (prothrombin time, activated partial thromboplastin time, and thrombin time) were performed using routine methods (Siemens Healthcare Diagnostic Products, Marburg, Germany). Fibrinogen concentrations were measured by the Clauss method. Commercially available ELISA tests were used to determine PAI-1 activity (Technozym PAI-1 Actibind, Technoclone, Vienna, Austria), plasmin-*α*_2_-antiplasmin (PAP) complex (Technozym PAP complex ELISA kit, Technoclone, Vienna, Austria), and thrombin-antithrombin (TAT) complex (Enzygnost TAT micro, Siemens Healthcare Diagnostic Products, Marburg, Germany). Factor VIII (FVIII) activity using a chromogenic assay, von Willebrand factor (VWF) antigen level, *α*_2_-plasmin inhibitor (*α*_2_-PI) activity, plasminogen activity and D-dimer levels were measured on a BCS coagulometer by standard methods (Siemens Healthcare Diagnostic Products, Marburg, Germany). Plasma levels of FXIII activity were determined by ammonia release assay [33] using a commercially available reagent kit (REA-chrom FXIII kit, Reanal-ker, Budapest, Hungary). Soluble fibrin monomer levels (FM) were measured using the Liatest FM assay (Diagnostica Stago, Asnières, France).

High sensitivity C-reactive protein (CRP) and a comprehensive lipid profile including total cholesterol, low-density lipoprotein (LDL) cholesterol, high-density lipoprotein (HDL) cholesterol, and triglyceride levels were measured from antecubital vein blood samples of all patients upon hospital admission by routine methods (Roche Diagnostics, Mannheim, Germany).

### 2.4. Statistical Analysis

All data were analyzed using the GraphPad Prism Software version 5.0 (La Jolla, CA) and the Statistical Package for Social Sciences (SPSS, Release 22.0, Chicago, IL). Normality of the data was evaluated by the D'Agostino and Pearson omnibus normality test. A paired* t*-test or Wilcoxon matched pairs rank-sum test was applied for comparing results obtained from intracardiac and FV samples. In case of two-group analyses between AF patients and controls, unpaired* t*-test or in case of nonparametric data Mann–Whitney *U* test was used. ANOVA or Kruskal-Wallis test was applied for multiple comparisons. Pearson's or Spearman's correlation coefficient was used to determine the strength of correlation between variables. Differences between categorical variables were assessed by the Fisher's exact test. *p* < 0.05 was considered statistically significant.

## 3. Results

### 3.1. Baseline Characteristics of AF Patients and Non-AF Controls

Clinical characteristics of the AF patient group and control group are shown in Table 1. In total 32 AF patients and 18 controls were enrolled in the study. Unfortunately, 8 AF patients and 4 controls had to be excluded from the study population due to technical problems arising during the intracardiac blood drawing procedure (clot formation in the sample during the blood drawing procedure, clot formation on the sheath requiring instant heparin administration, etc.). In case of 12 AF patients and 8 non-AF controls an LAA sample was not possible to obtain due to technical/anatomic difficulties. The final numbers of AF patients and non-AF controls included in the study were 24 and 14, respectively (Table 1). No significant differences were observed between the AF patients and non-AF controls regarding BMI and cerebrovascular risk factors except for smoking, which was more frequent in controls. Only two patients experienced paroxysmal AF periods during the procedure. Most AF patients had low or moderate risk for stroke according to the CHADS_2_ and CHA_2_DS_2_-VASC score. A similar fraction of AF patients and non-AF controls received statins and antihypertensive drugs. CRP levels and lipid parameters, measured from peripheral venous blood samples, did not differ significantly between AF patients and non-AF controls.

### 3.2. Intracardiac Levels of Hemostasis Factors in AF Patients and Non-AF Controls

**Figure 1:**
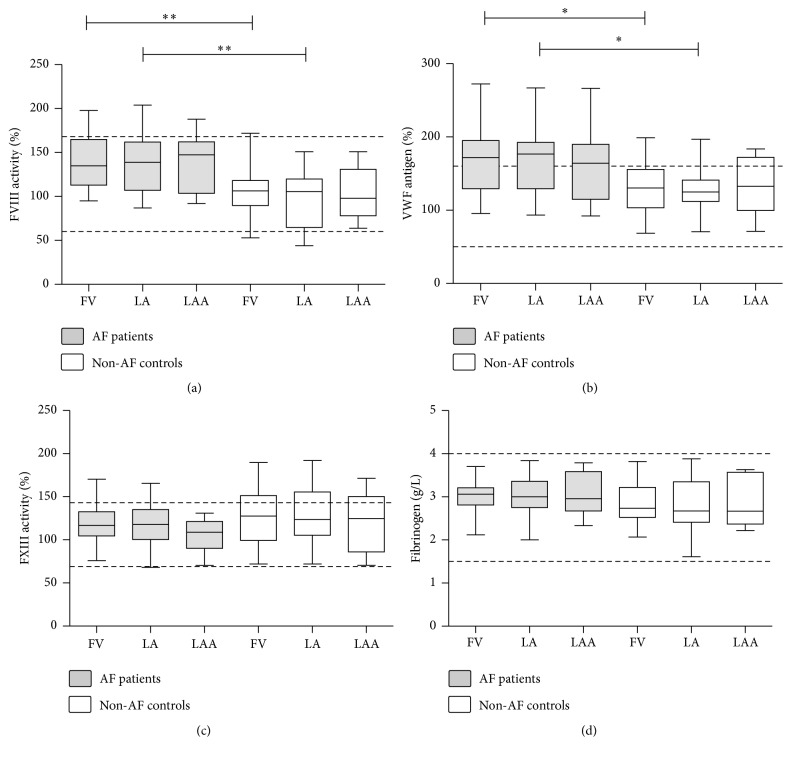
Levels of various coagulation factors in patients with atrial fibrillation (AF) and non-AF controls. Box and whisker plots indicate median, interquartile range, and total range. Dashed lines indicate upper and lower limits of reference interval. FVIII: factor VIII; VWF: von Willebrand factor; FXIII: factor XIII; FV: femoral vein; LA: left atrium; LAA: left atrial appendage. ^*∗*^*p* < 0.05; ^*∗∗*^*p* < 0.01.

FVIII activity and VWF antigen levels were significantly higher in the AF patient group as compared to the control group in the samples obtained from the FV and from the LA (Figure 1). LAA levels of both proteins showed a marked elevation in AF patients as well; however, very likely due to the lower number of LAA samples, results were only borderline significance. Elevated levels were not due to acute phase reaction as CRP levels of all individuals were in the normal range. In case of AF patients, median values of VWF antigen levels were above the upper limit of the reference interval in all sample types (171% (IQR: 129.4–195.1%), 176.7% (IQR: 129.3–192.7%), and 164% (IQR: 114.8–189.8%) for FV, LA, and LAA sample types, resp.). The observed differences between patients and controls remained significant after adjustments for AB0 blood type in the statistical model. No local differences were found in the FVIII and VWF levels of intracardiac samples as compared to the FV samples in either group. FVIII and VWF levels showed good correlation in AF patients (Spearman *r* = 0.808, 95% CI: 0.691–0.884, *p* < 0.0001) as well as in non-AF controls (Pearson *r* = 0.737, 95% CI: 0.502–0.871, *p* < 0.0001), suggesting that they are in a complex form. No considerable differences were seen in the correlation of FVIII and VWF levels with respect to sampling sites (data not shown). No significant differences were found between sample types and patient groups in case of FXIII activity and fibrinogen levels.

### 3.3. Intracardiac Levels of Coagulation Activation Markers in AF Patients and Non-AF Controls

**Figure 2:**
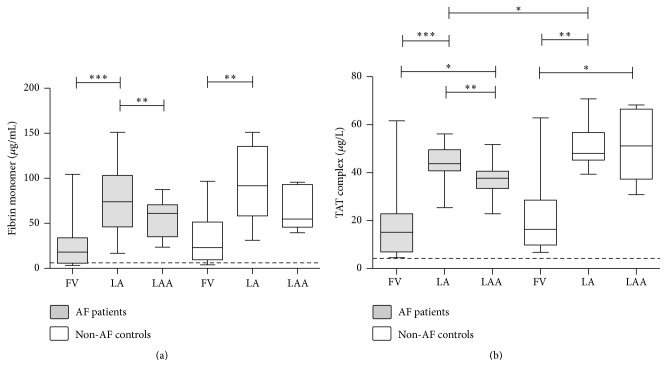
Levels of quantitative fibrin monomer and thrombin-antithrombin (TAT) complex in patients with atrial fibrillation (AF) and non-AF controls. Box and whisker plots indicate median, interquartile range, and total range. Dashed lines indicate upper limit of reference interval. FV: femoral vein; LA: left atrium; LAA: left atrial appendage. ^*∗*^*p* < 0.05; ^*∗∗*^*p* < 0.01; ^*∗∗∗*^*p* < 0.001.

Median values of soluble FM and TAT complex levels exceeded the upper limit of reference interval in the FV samples of AF patients (18.16 *μ*g/mL (IQR: 5.83–33.91 *μ*g/mL) and 15.17 *μ*g/L (IQR: 6.96–22.83 *μ*g/L) for FM and TAT, resp.) and non-AF controls (23.05 *μ*g/mL (IQR: 9.55–51.41 *μ*g/mL) and 16.36 *μ*g/L (IQR: 9.84–28.59 *μ*g/L) for FM and TAT, resp.) (Figures 2(a) and 2(b)). Moreover, both parameters were significantly elevated in the samples obtained from the LA as compared to the FV samples in case of both groups, suggesting that that the observed differences are not AF-specific and most probably the catheterization procedure itself has a major effect on the results. FM levels showed a decrease in the LAA samples as compared to the LA samples; this decrease was significant in case of the patients (*p* < 0.001, Wilcoxon matched pairs rank-sum test) (Figure 2(a)). TAT complex levels were also significantly lower in the LAA samples versus LA samples of AF patients (*p* < 0.01, Wilcoxon matched pairs rank-sum test), while such significant association was not observed in case of the non-AF control patients (Figure 2(b)). TAT complex levels were significantly increased in the LAA samples of both AF patients and non-AF controls as compared to the FV samples. Surprisingly, a marginal but significant elevation was observed in the TAT complex levels of the LA samples of non-AF controls versus AF patients (*p* < 0.05).

### 3.4. Intracardiac Parameters of Fibrinolysis in AF Patients and Non-AF Controls

**Figure 3:**
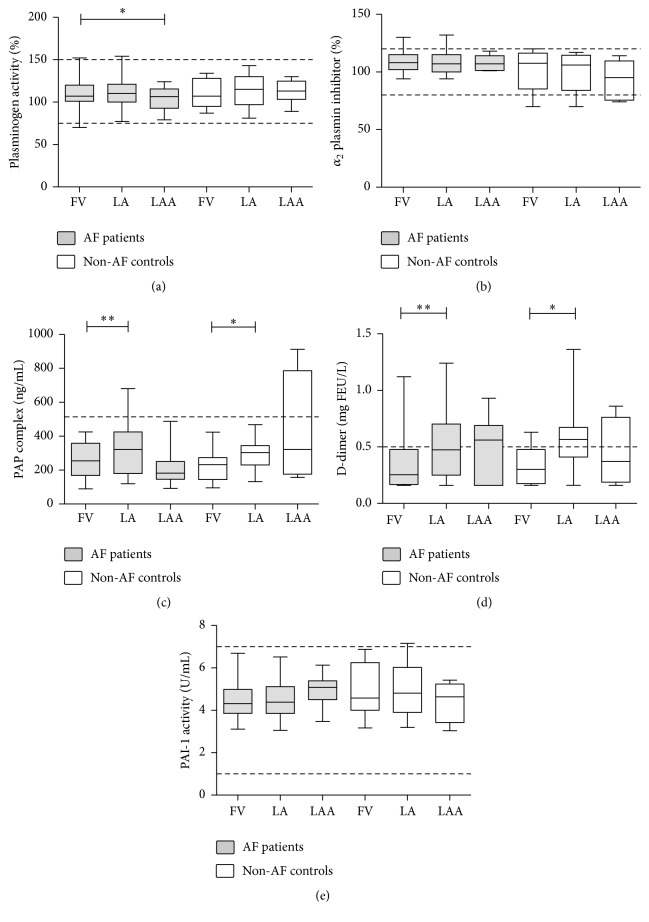
Levels of various fibrinolytic markers in patients with atrial fibrillation (AF) and in non-AF controls. Box and whisker plots indicate median, interquartile range, and total range. Dashed lines indicate upper and lower limits of reference interval or diagnostic cut-off levels. PAP complex: plasmin-*α*_2_-antiplasmin complex; PAI-1: plasminogen activator inhibitor type 1, FV: femoral vein; LA: left atrium; LAA: left atrial appendage; ^*∗*^*p* < 0.05; ^*∗∗*^*p* < 0.01.

Plasminogen activity, *α*_2_-PI activity, and PAI-1 activity levels showed no difference between AF patients as compared to non-AF controls (Figures 3(a), 3(b), and 3(d)). In general, no difference was observed between the intracardiac and peripheral levels of these parameters, except for a small, but significant reduction of plasminogen level in the LAA versus FV sample of the AF patients (Figure 1(a)). PAP complex and D-dimer levels were significantly increased in the LA samples of both AF patients and non-AF controls as compared to the respective FV samples (Figures 3(c) and 3(d)), suggesting that the activation of the fibrinolytic system took place during the transcatheter procedure in both groups. In fact, approximately half of the AF patients and non-AF controls had D-dimer levels exceeding the cut-off value in the LA sample, while median values of D-dimer were well below the cut-off in the FV samples (0.26 mgFEU/L (IQR: 0.17–0.48 mgFEU/L) and 0.30 mgFEU/L (IQR: 0.18–0.48 mgFEU/L) in AF patients and controls, resp.) (Figure 3(d)).

## 4. Discussion

Although it is a general belief that in AF the intracardiac milieu is more thrombogenic than the peripheral blood, supporting pieces of evidence derived from measurements using intracardiac blood samples are scarce. In this study, we investigated the levels of a comprehensive list of hemostasis and fibrinolysis markers from intracardiac blood samples of AF patients and non-AF controls and failed to detect significant AF-specific alterations of hemostasis or fibrinolysis in intracardiac blood samples. It is to be noted, however, that only two patients experienced paroxysmal AF periods during the procedure, which means that most patients were on sinus rhythm during blood sampling. Our results suggest that as compared to peripheral samples, paroxysmal/persistent AF patients have no significant alterations in the intracardiac levels of the investigated hemostasis and fibrinolytic parameters, at least when they are not experiencing AF periods.

Although significant local differences were observed for certain coagulation activation and fibrinolytic markers (namely, for FM, TAT complex, PAP complex, and D-dimer levels) in the intracardiac samples as compared to the FV samples, the same differences were found in non-AF control individuals. Moreover, in the LAA sample of both groups, a general tendency of decrease was observed in the level of most investigated markers as compared to LA samples. In earlier studies, in which non-AF control population was not investigated, these differences were attributed to AF pathophysiology [26]. However, our results imply that changes in the level of these markers are not specific for AF and are likely to be attributed to the invasive nature of the catheterization procedure, including transseptal puncture and tissue damage.

Among all investigated hemostasis and fibrinolysis parameters, only the elevation of FVIII and VWF levels was found to be AF-associated in our study. Interestingly, FVIII and VWF levels were significantly elevated in both peripheral and intracardiac blood samples of AF patients as compared to controls. Elevation of VWF levels was particularly considerable in the AF patient group as the medians of VWF levels were at the upper limit of the reference interval in all sample types. Although the levels of VWF in AF patients have been studied earlier using peripheral samples, the relationship between intracardiac and peripheral VWF levels has been obscure. An elevation of FVIII [18, 34] and VWF [35–38] has been described earlier in the peripheral samples AF patients and it has been proposed to be attributed to endothelial damage. Moreover, elevated levels of VWF have been associated with increased stroke risk and poor prognosis [36, 39, 40]. Only few papers enrolling a limited number of patients have investigated the levels of VWF in AF patients from both intracardiac and peripheral blood samples, but in these studies FVIII levels were not determined [27, 41]. In line with our findings, in these earlier reports it was found that VWF levels were similar in the intracardiac samples and in samples obtained from the peripheral sampling site. In our study FVIII and VWF levels showed good correlation in all sample types, suggesting that they were in complexed form. As both proteins are stored in the Weiber-Palade bodies of the endothelium [42], these results imply that the elevation of VWF and FVIII levels are the consequence of endothelial damage not necessarily restricted to the LA. It has to be noted that in the LAA of patients a similar tendency of FVIII and VWF elevation was observed as in case of FV and LA samples, but most likely due to the limited number of LAA samples, differences were not proved to be significant between patients and controls for this sample type.

Despite the important role of fibrinolytic system in preventing intravascular thrombosis, previous studies have paid little attention to the investigation of fibrinolytic abnormalities associated with AF [8]. Moreover, little is known about the levels of important regulators of fibrinolysis in intracardiac samples in AF. Here we assessed a series of fibrinolytic markers from both peripheral and intracardiac blood samples of AF patients and non-AF controls. Besides a small, but significant decrease in the levels of plasminogen in the LAA samples of AF patients as compared to the FV samples, no significant differences were observed between AF patients and non-AF controls and among sample types concerning FXIII activity, *α*_2_-plasmin inhibitor, PAI-1 activity, and plasminogen activity measurements. There was no difference between PAP complex and D-dimer levels in AF patients and non-AF controls as well. These finding suggests that the investigated components of the fibrinolytic system are mostly unaltered in AF.

## 5. Limitations

Our study has some limitations. First, the number of patients enrolled in the study was limited, which was obviously due to the highly invasive nature of the blood sampling, during which technical difficulties were often encountered. We would like to highlight, however, that the number of patients enrolled in our study is still more than the average number of patients undergoing this kind of blood sampling as published so far. Moreover, in our study a non-AF control patient group was also enrolled, which is often missing from earlier studies. Despite the particularly difficult and potentially risky technique of LAA sampling, a considerable number of patients were sampled from the LAA as well, which is a rarity in the literature as yet. Based on our findings larger studies are warranted to corroborate our observations.

Second, most patients enrolled in the study had low or moderate stroke risk according to the CHADS_2_ or CHA_2_DS_2_-VASC score, which limits the extrapolation of our findings to the general AF patient population. It has to be noted, however, that the stroke risk of our patient population reflects the current practice of most ablation centers, which offer ablation for younger patients with mostly paroxysmal AF, structurally normal heart, and no significant comorbidity [43]. In addition, the necessity and safety of the discontinuation of anticoagulation preablation (which was a requirement in our study in order to carry out certain measurements) are only evident in low-risk patients [44].

Third, only 2 patients experienced a paroxysmal AF period during the catheterization and blood drawing procedure. Naturally, more patients having AF period during sampling could have supplemented our results with a further interesting aspect.

## 6. Conclusion

AF patients have elevated FVIII and VWF levels, most likely due to endothelial damage, which is present in the intracardiac and peripheral environment as well. Intracardiac activation of hemostasis and fibrinolysis was demonstrated in AF patients and in non-AF controls to a similar extent, indicating that this might be a consequence of the catheterization procedure itself rather than a footprint of AF pathophysiology.

## Figures and Tables

**Table 1:** Characteristics of atrial fibrillation (AF) patients and controls.

Variables	AF group	non-AF control group	*p* value
Number of patients after exclusions	24	14	
Age (years)	56.45 (47.43–59.80)	50.60 (32.80–56.10)	0.061
Male, *n* (%)	15 (62.50)	10 (71.43)	0.728
BMI (kg/m^2^)	29.43 ± 5.52	26.10 ± 4.75	0.068
Cerebrovascular risk factors, *n* (%)			
Arterial hypertension	13 (54.17)	8 (57.14)	1.000
Hypercholesterolemia	19 (79.17)	8 (57.14)	0.266
Current smoking	2 (8.33)	6 (42.86)	0.013
Diabetes mellitus	2 (8.33)	0	—
Previous myocardial infarction, *n* (%)	0	0	—
Previous ischemic stroke, *n* (%)	0	0	—
Heart failure, *n* (%)	1 (4.17)	0	—
Left atrium size (mm)	39.71 ± 4.82	36.55 ± 4.80	0.081
AF period during procedure, *n* (%)	2 (8,33)	—	—
CHADS_2_ score, *n* (%)			
0	10 (41.67)	n.a.	—
1	10 (41.67)	n.a.	—
2	4 (16.67)	n.a.	—
CHA_2_DS_2_-VASC score, *n* (%)			
0	6 (25.00)	n.a.	—
1	9 (37.50)	n.a.	—
2	5 (20.83)	n.a.	—
3	3 (12.50)	n.a.	—
4	1 (4.17)	n.a.	—
EHRA score, *n* (%)			
1	3 (12.50)	n.a.	—
2	1 (4.17)	n.a.	—
3	16 (66.67)	n.a.	—
4	4 (16.67)	n.a.	—
Medication, *n* (%)			
Statin	6 (25.00)	2 (14.29)	0.684
ACEI	8 (33.33)	6 (42.86)	0.729
Beta-blocker	17 (70.83)	9 (64.29)	0.728
Laboratory parameters			
C-reactive protein (mg/L)	1.50 (0.60–2.75)	1.10 (0.13–2.05)	0.281
Total cholesterol (mmol/L)	5.30 ± 1.16	4.69 ± 1.09	0.122
LDL cholesterol (mmol/L)	3.42 ± 0.98	2.79 ± 0.91	0.061
HDL cholesterol (mmol/L)	1.44 (0.32)	1.46 ± 0.42	0.812
Triglyceride (mmol/L)	1.80 (1.23–2.18)	1.4 (0.75–1.95)	0.208
Zero blood group, *n* (%)	7 (29.17)	2 (14.29)	0.446

Continuous variables are expressed as mean ± SD or median (interquartile range). Categorical variables are indicated as number (percentage), unless otherwise stated. AF: atrial fibrillation; *n*: number; IQR: interquartile range; SD: standard deviation; n.a.: nonapplicable; BMI: body mass index; CHADS_2_ score: congestive heart failure, hypertension, age ≥ 75 years, diabetes mellitus, stroke/transient ischemic attack; CHA_2_DS_2_-VASC score: congestive heart failure, hypertension, age ≥ 75 years, diabetes mellitus, stroke/transient ischemic attack/thromboembolism, vascular disease (prior myocardial infarction, peripheral vascular disease, or aortic atherosclerosis), age (65–74 years), sex category (female); EHRA score: European Heart Rhythm Association score; ACEI: angiotensin converting enzyme inhibitor; LDL cholesterol: low density lipoprotein cholesterol; HDL cholesterol: high density lipoprotein cholesterol.
